# Direct-write nanoscale printing of nanogranular tunnelling strain sensors for sub-micrometre cantilevers

**DOI:** 10.1038/ncomms12487

**Published:** 2016-09-26

**Authors:** Maja Dukic, Marcel Winhold, Christian H. Schwalb, Jonathan D. Adams, Vladimir Stavrov, Michael Huth, Georg E. Fantner

**Affiliations:** 1 Laboratory for Bio- and Nano-Instrumentation, Interfaculty Institute of Bioengineering, École Polytechnique Fédérale de Lausanne, Batiment BM 3109 Station 17, 1015 Lausanne, Switzerland; 2 Physikalisches Institut, Max-von-Laue-Street 1, Goethe-Universität, 60438 Frankfurt am Main, Germany; 3 NanoScale Systems, Nanoss GmbH, Robert-Bosch-Street 7, 64293 Darmstadt, Germany; 4 AMG Technology Ltd., Microelectronica Industrial Zone, 2140 Botevgrad,Bulgaria

## Abstract

The sensitivity and detection speed of cantilever-based mechanical sensors increases drastically through size reduction. The need for such increased performance for high-speed nanocharacterization and bio-sensing, drives their sub-micrometre miniaturization in a variety of research fields. However, existing detection methods of the cantilever motion do not scale down easily, prohibiting further increase in the sensitivity and detection speed. Here we report a nanomechanical sensor readout based on electron co-tunnelling through a nanogranular metal. The sensors can be deposited with lateral dimensions down to tens of nm, allowing the readout of nanoscale cantilevers without constraints on their size, geometry or material. By modifying the inter-granular tunnel-coupling strength, the sensors' conductivity can be tuned by up to four orders of magnitude, to optimize their performance. We show that the nanoscale printed sensors are functional on 500 nm wide cantilevers and that their sensitivity is suited even for demanding applications such as atomic force microscopy.

## Abstract (web summary)

Reducing the size of cantilever-based sensors increases the sensitivity and detection speed of techniques such as atomic force microscopy. Here, the authors demonstrate a nanomechanical readout method that can be easily scaled down in size by using electron co-tunnelling through a nanogranular metal.

## Graphical abstract

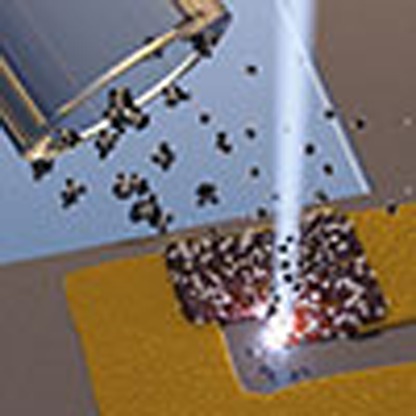

Micro- and nanomechanical sensing is widely used in atomic force microscopy (AFM) nanocharacterization^1^, biosensing^2^,^3^, mechanical property measurements^4^, and force^5^,^6^ and mass sensing^7^,^8^,^9^. The devices used generally consist of a micro-electro-mechanical systems (MEMS)-based force sensor, often a cantilever, in combination with a method to detect its deflection. Resonant sensors are very prevalent due to their high sensitivity. The sensitivity of the measurement depends both on the mechanical properties and geometry of the cantilever, as well as on the signal-to-noise ratio (SNR) of the deflection detection method. Decreasing all three dimensions of the cantilever to the sub-micrometre range drastically decreases its inertial mass and thereby increases its sensitivity, resonance frequency and detection bandwidth^10^,^11^. Such miniaturization has recently pushed the limits in nanoscale processes studies, enabling video rate imaging in high-speed AFM (HS-AFM)^11^,^12^,^13^. However, further decrease in size rules out traditional optics-based approaches to measure the cantilever deflection, as the cantilever dimensions are below the conventional optical detection limit. Strain sensors integrated with the cantilever can circumvent the problems with optical detection. These sensors can be piezoelectric^14^ or piezoresistive^5^,^15^ in nature. However, a fundamental problem for using these sensing materials on very small cantilevers is the minimum required size and especially thickness of the sensor elements (hundreds of nanometres to micrometres)^16^,^17^. This often exceeds the total allowable thickness of these cantilevers, typically <100 nm. Li *et al.*^10^ have demonstrated the sensing of impressively small cantilevers using very thin metal film sensors (30 nm) by measuring the change in resistance due to geometric effects. With such approaches however, the resistance of the sensor is often much less than the resistance of the connecting leads, which can significantly reduce the effective signal (Supplementary Figs 1–2; Supplementary Table 1 Supplementary Note 1). In the past nanogranular metals with tens of micrometre length have shown exceptional promise in terms of sensitivity, ease of deposition and versatility of substrate materials in static strain-sensing applications of large MEMS structures (500 μm long cantilevers)^18^.

Here we present the use of direct-write printing of nanogranular metals to write nanometre-sized nanogranular tunnelling resistor (NTR) strain sensors onto prefabricated cantilevers (Fig. 1a). The tunnelling nature of the electron transport in the nanogranular metal results in a highly tunable, sensitive strain gauge with thicknesses down to 5 nm. The unique benefit of such deposited NTRs lies in their opportunity for the miniaturization down to the tens of nanometre range, which makes them suitable for dynamic sensing of sub-micron-sized mechanical devices. We demonstrate that NTR sensors can be used for the deflection sensing of sub-micrometre cantilevers and that their sensitivity is well suited even for demanding applications such as amplitude modulation AFM.

## Results

### Nanoscale printing of NTR sensors

**Figure 1:**
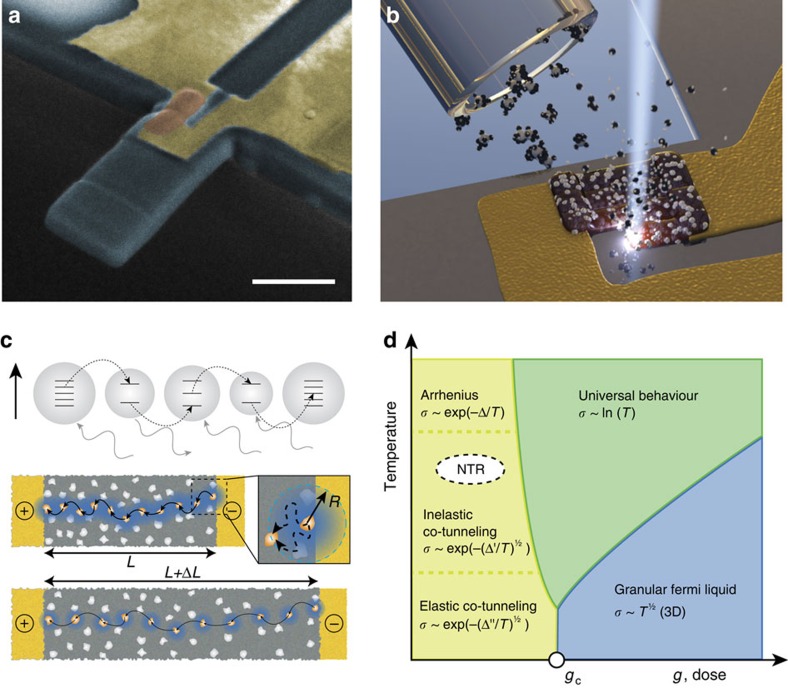
Fabrication and electronic transport in nanogranular tunnelling resistor sensors. (**a**) Example of nanogranular tunnelling resistor (NTR) deposited on a 1 μm wide, 300 nm thick free standing SiN cantilever. The scanning electron microscopy (SEM) image is false coloured to distinguish the NTR sensor (orange) and the metal contacts (yellow). Scale bar, 1 μm. (**b**) Illustration of the NTR electron-beam-induced deposition process. Precursor gas molecules adsorb and diffuse on the surface, where they are dissociated by the scanned electron beam and form platinum clusters embedded in a carbonaceous matrix. (**c**) Schematic depiction of the inelastic co-tunnelling process in the NTR. Electrons tunnel through several grains at the same time via virtual energy levels. The co-tunnelling radius therefore is larger than the inter grain distance (blue halos). When the sensor is stretched, the inter grain distance increases and the co-tunnelling radius decreases that results in an increased resistance. (**d**) Phase diagram of the electronic transport regimes in granular metals. This phase diagram was theoretically predicted based on a recent theoretical investigations^24^ and was largely verified experimentally by very recent experiments on Pt(C)-based granular metal samples with finely tuned tunnel coupling *g* (ref. 25). The conductivity temperature dependence is given for each regime, where Δ′–Δ′′′ are temperature constants that depend on the NTR material properties and conduction regime^22^. At room temperature and the prevailing coupling strength, the NTR sensors operate within the inelastic co-tunnelling regime.

The NTR sensors are deposited using focused electron-beam-induced deposition (FEBID). For this, a platinum-based gaseous precursor [MeCpPt(Me)3] is introduced in the vicinity of the focal spot of a scanning electron microscope (Fig. 1b). The precursor molecules adsorb on the surface and are dissociated in the focus of the scanned electron beam^19^. The freed platinum atoms form clusters or nanoparticles that become embedded in a matrix of deposited carbon atoms. The Pt(C) NTRs are composed of 22–23 at% Pt and 77–78 at% C, in form of platinum nanocrystallites with a diameter of 2–5 nm that are embedded in a dielectric, carbonaceous matrix. The NTR sensor can be deposited on almost any material and, due to the excellent depth of focus, on almost arbitrarily shaped surfaces.

### Tuning of charge transport mechanism

The charge transport in NTR sensors occurs through the tunnelling between the Pt nanoparticles. This tunnelling process strongly depends on the Coulomb charging energy *E*_C_ of the nanoparticles, and the inter-granular tunnel-coupling strength *g*. *E*_C_ is largely determined by the capacitance *C* of the nanoparticles (in the dielectric matrix) and therefore their size. *g* is dominated by the properties of the dielectric matrix and the particle-to-particle distance. The exponential dependence of the tunnelling probability on the inter-granular distance suggests the suitability of the material as a high-sensitivity strain sensor. When the material is strained in tension, the average distance between the particles increases, the tunnelling probability decreases and the resistivity of the NTR increases. Previous attempts to use tunnelling as strain-sensing readout have focused on the integration of single tunnel junctions or single electron transistors onto cantilevers^20^,^21^. In NTR sensors, however, multiple tunnelling events are involved in the electron transfer through the whole resistor (represented by the chain of solid arrows in Fig. 1c). Transport mechanisms in nanogranular metal can differ significantly due to the wide possible range of the inter-granular-coupling strength. This results in a phase diagram of the transport regimes, as is schematically depicted in Fig. 1d. The regime of activated transport or correlated variable range hopping (dashed region in Fig. 1d) is most relevant for the strain-sensing effect in the cantilever sensors^22^. In this regime, the Coulomb blockade of each single nanoparticle is partially lifted by charged defects surrounding it^23^. As the nanoparticle size gets smaller, the charging energy grows like *E*_C_∼1/*C*. As a consequence, charge transport is dominated by thermally assisted tunnelling between nanoparticles in a way that the electrostatic energy of the charge carriers along the tunnelling path is kept small. Theory suggests that for these NTR materials at room temperature each individual electron transfer event is a co-tunnelling event, where electrons can hop over distances larger than the average distance between two nanoparticles^24^. This tunnelling process can occur through tunnelling via virtual electron levels in a sequence of nanoparticles (dotted arrows in Fig. 1c). The result is a co-tunnelling radius *r* shown in blue. Due to their small size, the nanoparticles exhibit discrete quantum mechanical energy levels inside the grains. During co-tunnelling, the energy level of the starting particle and the energy level of the final particle can be different. The electron therefore creates electron–hole excitations, as it tunnels out of the virtual intermediate state, making the co-tunnelling inelastic. From this theoretical description of the electron transport process, we can derive guidelines for optimization of the NTR material by tuning the deposition and post processing conditions.

**Figure 2:**
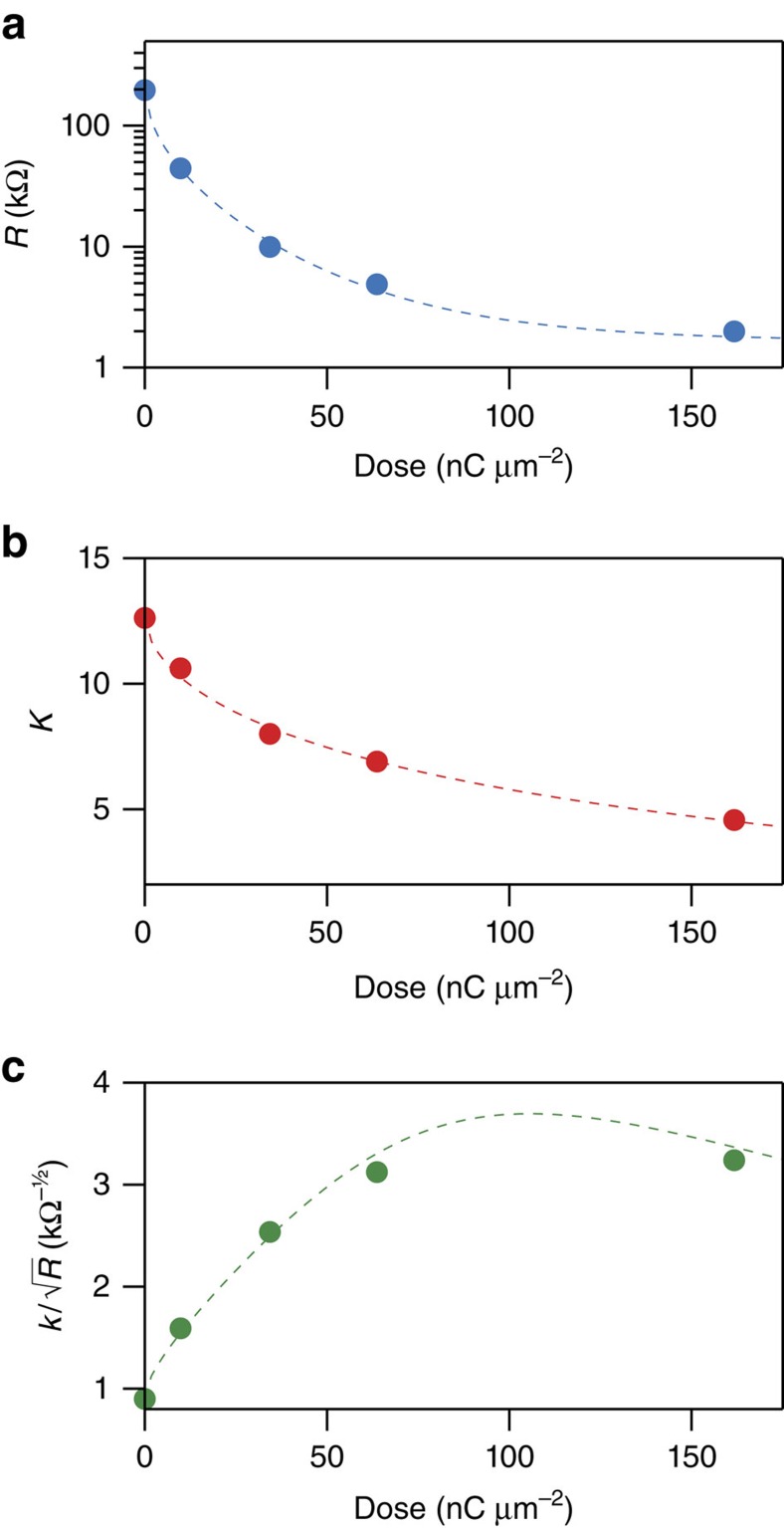
Signal-to-noise ratio optimization of nanogranular tunnelling resistor sensors. (**a**) Measured reduction of resistance *R* of typical nanogranular tunnelling resistor (NTR) strain sensor element (active sensor element size 4 × 2 × 0.2 μm^3^) versus effective post-growth electron irradiation dose. Within the applied dose range transport remains in the inelastic co-tunnelling regime (see dashed ellipse in Fig. 1d). (**b**) Associated measured drop of NTR gauge factor *κ* versus irradiation dose. (**c**) Calculated dose-dependent signal-to-noise ratio as a consequence of its *κ/R*^1/2^ dependence given in equation (1). The dashed lines serve to guide the eye.

Post-growth electron irradiation is an efficient and fast method to increase the inter-granular tunnel-coupling strength *g*, since it leads to a transformation of the carbon matrix from amorphous carbon to nanocrystalline graphite, resulting in a strong reduction of the resistivity of the strain sensor elements^25^,^26^,^27^. This is shown in Fig. 2a for a typical sensor. As the irradiation dose is increased, the resistance of the sensor elements drops by two orders of magnitude as a function of dose *d* in the relevant transport regime. As predicted, the gauge factor of the strain sensor drops as well (Fig. 2b). Here we use the observed dose dependence of resistance and gauge factor to optimize the SNR achievable with NTR-based strain sensors on very small cantilevers. We define the SNR as the ratio of the cantilever peak thermomechanical fluctuations *A*_max_ to the NTR voltage noise *n*_*J*_, scaled by the deflection sensitivity DS:


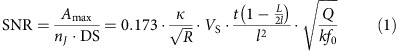


factorized in NTR sensor element specific aspects (*κ*: gauge factor, *R*: resistance, *V*_S_: supply voltage to the Wheatstone bridge) and a geometry/material factor governed by the cantilever dimensions (*L*: NTR sensor length, *l* and *t*: cantilever length and thickness) and its oscillator properties (*Q*: quality factor, *k*: spring constant and *f*_0_: resonance frequency). For details see Supplementary Note 2. Figure 2c shows the NTR-specific sensitivity factor 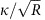 as a function of the post-growth irradiation dose. The sensitivity increases by more than a factor of 3 for low doses. A rather broad sensitivity maximum occurs for dose values between 75 and 200 nC μm^−2^, which results in the reproducible sensor performance.

### Sensor scalability

**Figure 3:**
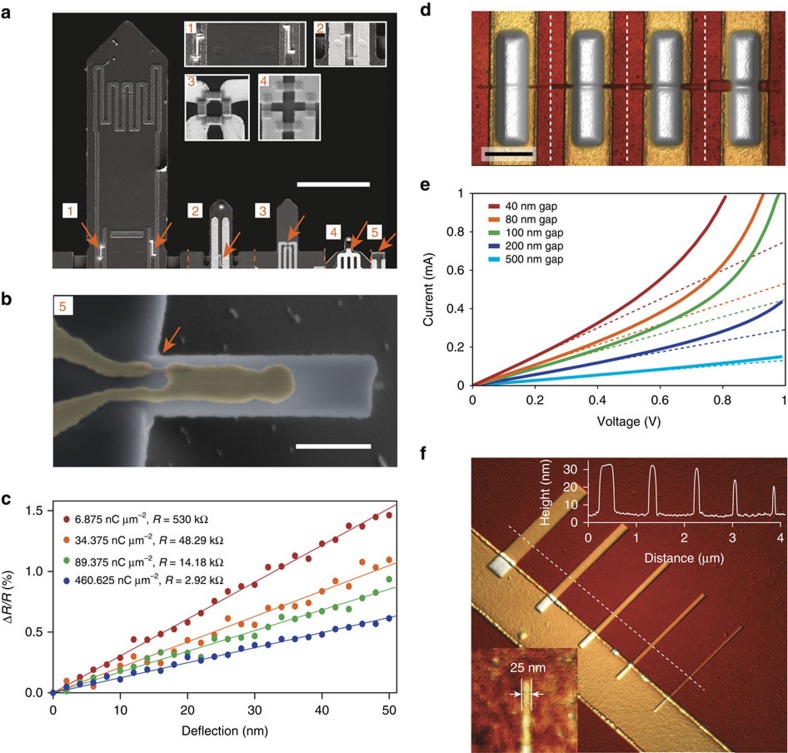
Scalability of nanogranular tunnelling resistor sensors. (**a**) Nanogranular tunnelling resistor (NTR) sensors deposited on a variety of custom-made atomic force microscopy (AFM) cantilevers spanning two orders of magnitude. Scale bar, 100 μm. (**b**) Scanning electron microscopy image of a 1.5 × 0.5 × 0.1 μm^3^ cantilever with NTR sensor. Scale bar, 500 nm. (**c**) Dose-dependent deflection curves of a 500 nm wide cantilever (see gauge factor estimates in Supplementary Figure 8 and Supplementary Note 7). (**d**) AFM images of NTR sensors with different active sensor lengths. The active sensor length is determined only by the electrode gap, which can go down to 40 nm. Scale bar, 1 μm. (**e**) *I*/*V* curves of un-strained NTR sensors for different active sensor lengths. E-beam irradiation dose used after sensor deposition was 74 nC μm^−2^. Dashed lines serve to guide the eye and present sensor *I*/*V* dependence in the linear regime. At higher voltages, the onset of non-linearity is due to the overheating effects (Supplementary Figs 3–4; Supplementary Note 3). (**f**) Scalability of NTR deposition in width and height. Dimensions of down to 20 nm in width and 5 nm in height can be achieved routinely. The achievable resolution here is primarily determined by the substrate roughness.

The tunability of the electrical properties together with the flexibility of the sensor rapid prototyping method makes it possible to use the same sensing concept for cantilevers that vary two orders of magnitude in size. Figure 3a shows a montage of different size cantilevers equipped with NTR sensors (see orange arrows and inset figures), with cantilever widths down to 500 nm (Fig. 3b). Representative deflection curves of such a 500 nm wide cantilever are shown in Fig. 3c (Supplementary Movie 1). Much of the performance of the self-sensing cantilever depends not only on the material-specific performance of the sensor, but on the exact position and size of the strain-sensing element^28^. The precision of the nanoscale deposition can therefore be used to obtain the optimal sensitivity. In addition, optimal performance of a resistive strain sensor on a cantilever is reached when the active length of the sensor (that is, the gap between the electrodes) is as short as possible without compromising the electron transport mechanism. This is because the sensor length influences the resistance and thereby the Johnson noise. The direct-write nanoscale fabrication of the NTR sensors allows for extreme flexibility in sensor length, thickness and width. By controlling the gap size between the electrodes before deposition, we have achieved active sensor lengths down to 40 nm (Fig. 3d, leftmost). In such short active sensor lengths, only 10–15 nanoparticles in a direct chain are involved in the strain-sensitive electron transport (for grain sizes of ∼2–3 nm at 0.5 nm peripheral distance). Still, the *I*/*V*-characteristic for different active sensor lengths (Fig. 3e), shows linear behaviour up to a threshold electric field of at least 15 kV cm^−1^, beyond which local heating effects lead to the deviation from linearity (see Supplementary Figs 3–4 and Supplementary Note 3 for details).

Minimizing the thickness and width of the sensing element is also crucial to maintain a high signal from a strain-sensing element in a nano-cantilever. The sensor should be thin enough as to not contribute much to the flexural rigidity of the cantilever. This is especially problematic for nano-cantilevers intended for the measurement of small forces at high bandwidths (such as cantilevers for HS-AFM). Cantilevers for the next-generation HS-AFM will require total thicknesses <50–100 nm and width below 500 nm, which rules out the use of sensors made of conventional self-sensing materials such as doped Si, PZT or AlN. One of the most extraordinary features of our NTR sensors is the scalability of the sensor in height and width, down to single nanoparticles in height and 10 nm in width. Figure 3f shows NTR sensors deposited with varying thicknesses and widths. The inset shows a continuous sensor having a width of 25 nm and a thickness of 15 nm, which is sufficiently small for cantilevers down to a thickness of 40 nm and a width of 200 nm. The ability to deposit such extremely small-sized strain elements is an essential advantage of FEBID-deposited NTR sensors over other strain-sensing methods based on discontinuous metal layers^29^,^30^. The small size and thickness enables the fabrication of strain sensors on small AFM cantilevers without greatly affecting their dynamic properties (Supplementary Fig. 5; Supplementary Note 4). The achievable small active sensor length is essential for maintaining small resistances and a high detection bandwidth.

### Performance characterization

**Figure 4:**
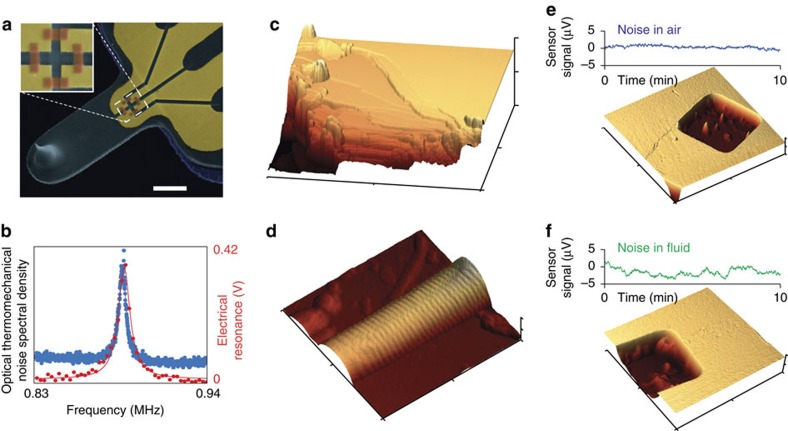
Atomic force microscopy imaging using nanogranular tunnelling resistor active readout. (**a**) Scanning electron microscopy image of the SiN atomic force microscopy (AFM) cantilever for dual readout (dimensions: 20 × 8 × 0.3 μm^3^). Inset: nanogranular tunnelling resistor (NTR) full bridge. Scale bar, 5 μm. (**b**) Driven resonance curve measured with NTR sensor (red) and optical thermal spectrum (blue). (**c**) AFM image of atomically flat steps on scratched mica. Total scan size: 5 × 5 μm^2^. Topography height: 1 μm. (**d**) AFM image of rat-tail collagen fibril showing the characteristic 67 nm spaced, 6 nm high banding pattern. Total scan size: 2.1 × 2.1 μm^2^. Topography height: 370 nm. (**e**,**f**) Noise of NTR sensor in air (**e**) and PBS (**f**) with AFM image of a silicon calibration grating obtained in air (**e**) and fluid (**f**). Total scan size for both images: 10 × 10 μm^2^. Topography height: (**e**) 270 nm and (**f**) 220 nm.

To test the functionality of the NTRs in a sensing application we have equipped a small, high-speed silicon nitride (SiN) AFM cantilever with a full Wheatstone bridge of NTRs (Fig. 4a). The full Wheatstone bridge readout cancels any NTR temperature-dependent effects, such as the influence of temperature on the NTR conductivity (with the conductivity temperature dependence given in Fig. 1d). The active sensor length used was 500 nm. The dimensions of the AFM cantilevers (20 × 8 × 0.3 μm^3^) were chosen so that it was still possible to use a laser for comparison readout (see Supplementary Fig. 6 and Supplementary Note 5 for details). Figure 4b shows the driven resonance curve measured with the NTR sensor (red). The optically detected thermomechanical tune is shown for reference (blue). The quality factor of alike cantilevers in air is between 100 and 200 depending on fabrication tolerances and tip length. Figure 4c,d shows tapping mode images of a rat-tail collagen fibre and atomically flat terraces in mica, respectively. The collagen fibre shows the characteristic 67 nm periodic banding pattern. The image quality proves the applicability of the NTRs as strain sensors even for demanding applications such as AFM. However, in these measurements the noise floor of the electrically detected deflection was not thermomechanically limited due to the still relatively large cantilever size. At this cantilever size, optical detection methods are still superior since they measure angular deflection rather than strain (Supplementary Fig. 7; Supplementary Table 2; Supplementary Note 6). Self-sensing cantilevers having noise levels comparable to optical readout were previously reported^10^ for cantilever dimensions in the sub micron range. The fully released cantilevers with a sharp tip we used for AFM imaging were 20 × 8 × 0.3 μm^3^, corresponding to the large cantilevers reported in ref. 10 (with lengths in 10 s of μm) . The noise levels of the cantilevers of similar dimension are similar between NTR and thin gold. In the same manner, with smaller cantilever dimensions the NTR performance improves. Below a cantilever length of ∼3 μm (thickness 300 nm), the NTR sensors are expected to outperform optical detection given reasonably achievable electronic noise levels (Supplementary Fig. 7). At lengths <1 μm, traditional optical lever deflection readout is no longer possible due to the diffraction limit^31^,^32^. An additional significant advantage compared with purely metallic strain sensors arises from the tunability of the NTR resistance by means of post-growth irradiation. Even for active sensors lengths <50 nm, the NTR resistance can be adjusted in the kΩ regime. As a result, degradation of the SNR due to the parasitic resistances of the connecting leads becomes negligible (Supplementary Figs 1–2; Supplementary Table 1; Supplementary Note 1). Moreover, if NTRs are applied for resonator sensing, the SNR is not as affected by the low frequency ‘flicker' noise, usually associated with low carrier density sensors.

The small active sensor lengths also allow for the use of small bridge voltages of 0.1–0.5 V, while still giving sufficient readout signal. This in principle enables the use of the NTR sensors for measurements in fluid even without passivation. Figure 4e,f shows the comparison of the sensor noise of a cantilever operated in air versus phosphate-buffered saline (PBS). NTRs were tested in PBS for several hours and remained stable. However, the noise fluctuations in PBS were a factor of 2 higher than in air. We hypothesize this is due to the interaction of ions with the surface of the NTR. So far we do not have sufficient data for a comprehensive description of the influence of different ionic strengths on the detection noise. However, conductance modulation effects of Pt(C) nanogranular metals immersed in polarizable media were theoretically investigated in the past^33^,^34^. The developed models are in agreement with our measurements, predicting time-dependent conductance modulations that are reflected as an increase of the NTR sensor noise. According to the theory, conductance modulations result from the interaction of ions with the surface of the NTR, changing the effective dielectric properties of the medium adjacent to the NTR. This effect could be reduced through passivation of the NTR sensor element. Nevertheless, even without passivation the NTR sensors remained stable in PBS for hours and we did not observe any degradation of the sensor.

## Discussion

The NTR direct-write nanoscale printing technology is extremely flexible in size, shape, substrate material, as well as sensor operating environment. This makes it possible, for the first time, to add strain sensing on very small and thin devices, and on unconventional materials and shapes, and opens the door for a range of applications, from sensing of nanowire cantilevers for HS-AFM to various three-dimensional (3D) resonators^35^. The deposition, however, is a serial process, where the sensors are written one at a time with an electron beam. The writing time for one sensor depends strongly on the size and is in the order of 2 min for our current full bridge set-up of NTR sensors (Fig. 4a) with an additional irradiation time of 1.5 min. For the smallest cantilevers shown in Fig. 3b, deposition as well as irradiation of an NTR can be realized in <10 s. We have successfully implemented a semi-automated deposition of the NTR sensors, which makes larger scale fabrication viable (Supplementary Fig. 9; Supplementary Note 8; Supplementary Movie 2).

While we have shown in this paper the use of NTR sensors for AFM cantilever sensing, the same sensor and rapid prototyping technology can also be used in cantilever-based bio sensors, 3D MEMS and nano-electro-mechanical systems (NEMS) devices, as well as strain-field sensing on flexible membranes such as those currently used in neural interfaces^36^. Future research will focus on optimizing the gauge factor by investigating alternative metal pre-cursors and dielectrics, and deposition of NTR sensors on chemical vapour deposition grown Si nanowires^37^ for use as ultra-HS-AFM cantilevers. These cantilevers can have mechanical resonance frequencies two orders of magnitude larger (100 MHz) than cantilevers currently used in HS-AFM. Such cantilever frequencies will be required for imaging the function of many molecular machines, which operate at timescales of 5 ms or less^12^.

## Methods

### NTR deposition and irradiation

The NTR strain sensors were fabricated by FEBID using the precursor trimethylmethylcyclopentadienylplatinum(IV) [MeCpPt(Me)3] and a dual-beam scanning electron microscope/focused ion beam (FIB) microscope (FEI, Nova Nanolab 600) equipped with a Schottky electron emitter with an ultimate resolution of 1 nm. The microscope is equipped with a gas injection system, which introduces the precursor gas via a 0.5 mm diameter capillary in close proximity to the focus of the electron beam. An electron beam energy of 5 keV and an electron current of 1.1 nA were employed for the FEBID process using s-shaped stripe-like patterns (serpentine scanning mode) that were repeatedly rastered over the structure at fixed dwell time per pixel (1 μs) and pitch (20 nm) between pixels. The post-growth electron irradiation was performed by using electron beam energy of 5 keV, electron current of 2.6 nA, dwell time of 10 μs and pitch of 20 nm.

### Static deflection and gauge factor measurements

The gauge factor measurements were performed by deflecting the NTR cantilever with a closed loop nanomanipulator system (SMARACT SLC1720-SL) integrated inside the electron microscope. During the deflection measurements the relative change in resistance due to the deflection was measured using a lock-in amplifier (Stanford Research SR830) and a Wheatstone bridge set-up. To achieve sub-nm resolution for the deflection of the smallest cantilevers shown in Fig. 3b, the cantilevers were mounted onto a piezo and pushed against the overlying nanomanipulator needle (Supplementary Movie 1).

### AFM cantilever fabrication

The self-sensing AFM cantilevers were fabricated using standard microfabrication techniques. Contacts to the NTRs were made with Cr/Au traces. On the 20 × 8 μm^2^ cantilevers, the fine-scale electrical contact structures were defined in the Cr/Au traces using focused ion-beam milling.

The submicron-width cantilevers were made starting with electron-beam lithography and dry etching to define the SiN cantilever shape on a silicon wafer. Electron beam and photolithography along with lift-off methods defined the Ti/Au metal contacts. The cantilevers were released with a short anisotropic silicon etch. In both cases, the NTR sensor was afterwards deposited to bridge the gap between the Au metal contacts, partially covering them.

### NTR readout instrumentation

We used a Wheatstone bridge configuration to measure the NTR resistance change. Initial amplification was achieved via a low-noise instrumentation amplifier positioned close to the cantilever chip to minimize the noise and stray capacitance. Subsequent amplification stages and the rest of the readout electronics were placed in a separate module designed to interface with a commercial AFM (Bruker MultiMode). The −3 dB bandwidth of the readout electronics at 40 dB gain was 2 MHz for a 1 kΩ bridge resistance.

### AFM measurements

All AFM imaging was performed in tapping mode using a commercial AFM system (Bruker MultiMode or Anfatec DS4). The NTR readout electronics were interfaced to either the standard MultiMode optical readout head, a custom-made MultiMode-compatible optical readout head for small cantilever deflection detection or a custom-built Anfatec DS4-compatible tip scanner. The cantilever resonances were excited using sheet or stack piezoactuators. Sample preparation and imaging conditions were as follows: the mica sample was lightly scratched with 1,000 grit sandpaper and cleaned with a CO_2_ snow cleaner. The AFM image was taken in air at 1.5 Hz scan rate and at 323 kHz resonance frequency. We obtained collagen from a rat-tail tendon sample. The tendon was placed on a freshly cleaved mica disk immersed in deionized (DI) water. The tendon was pulled apart using sharp tweezers to spread the individual fibres across the mica disk and left to dry at room temperature. The AFM image was taken in air at 0.5 Hz scan rate and at 419 kHz resonance frequency. Images of the silicon calibration grating (Bruker STR 10-1800P) were taken at 1 Hz scan rate and 913 kHz resonance frequency in air, and 332 kHz resonance frequency in water.

### Data availability

All data supporting the findings of this study are available from the authors. The authors declare that the data supporting the findings of this study are available within the article and its Supplementary Information files.

## Additional information

**How to cite this article:** Dukic, M. *et al.* Direct-write nanoscale printing of nanogranular tunnelling strain sensors for sub-micrometre cantilevers. *Nat. Commun.* 7:12487 doi: 10.1038/ncomms12487 (2016).

## Supplementary Material

Supplementary Information Supplementary Figures 1-9, Supplementary Tables 1-2, Supplementary Notes 1-8 and Supplementary References

Supplementary Movie 1 Deflecting a sub-micron cantilever using a piezo stage and a nanomanipulator needle

Supplementary Movie 2 The semi-automated process of nanogranular tunneling resistor deposition

## References

[b1] Giessibl F. J. Advances in atomic force microscopy. Rev. Mod. Phys. 75, 949–983 (2003).

[b2] Huber F., Lang H. P. & Gerber C. Nanomechanical sensors: Measuring a response in blood. Nat. Nanotechnol. 9, 165–167 (2014). 24584273 10.1038/nnano.2014.42

[b3] Huber F., Lang H. P., Backmann N., Rimoldi D. & Gerber C. Direct detection of a BRAF mutation in total RNA from melanoma cells using cantilever arrays. Nat. Nanotechnol. 8, 125–129 (2013). 23377457 10.1038/nnano.2012.263

[b4] Herruzo E. T., Perrino A. P. & Garcia R. Fast nanomechanical spectroscopy of soft matter. Nat. Commun. 5, 3126 (2014). 24445593 10.1038/ncomms4126

[b5] Arlett J. L., Maloney J. R., Gudlewski B., Muluneh M. & Roukes M. L. Self-sensing micro- and nanocantilevers with attonewton-scale force resolution. Nano Lett. 6, 1000–1006 (2006).

[b6] Rugar D., Budakian R., Mamin H. J. & Chui B. W. Single spin detection by magnetic resonance force microscopy. Nature 430, 329–332 (2004). 15254532 10.1038/nature02658

[b7] Ilic B. *et al.* Attogram detection using nanoelectromechanical oscillators. J. Appl. Phys. 95, 3694–3703 (2004).

[b8] Burg T. P. *et al.* Weighing of biomolecules, single cells and single nanoparticles in fluid. Nature 446, 1066–1069 (2007). 17460669 10.1038/nature05741

[b9] Feng X. L., He R., Yang P. & Roukes M. L. Very high frequency silicon nanowire electromechanical resonators. Nano Lett. 7, 1953–1959 (2007).

[b10] Li M., Tang H. X. & Roukes M. L. Ultra-sensitive NEMS-based cantilevers for sensing, scanned probe and very high-frequency applications. Nat. Nanotechnol. 2, 114–120 (2007). 18654230 10.1038/nnano.2006.208

[b11] Kodera N., Yamamoto D., Ishikawa R. & Ando T. Video imaging of walking myosin V by high-speed atomic force microscopy. Nature 468, 72–76 (2010). 20935627 10.1038/nature09450

[b12] Uchihashi T., Iino R., Ando T. & Noji H. High-speed atomic force microscopy reveals rotary catalysis of rotorless F1-ATPase. Science 333, 755–758 (2011). 21817054 10.1126/science.1205510

[b13] Fantner G. E., Barbero R. J., Gray D. S. & Belcher A. M. Kinetics of antimicrobial peptide activity measured on individual bacterial cells using high-speed atomic force microscopy. Nat. Nanotechnol. 5, 280–285 (2010). 20228787 10.1038/nnano.2010.29 PMC3905601

[b14] Karabalin R. B. *et al.* Piezoelectric nanoelectromechanical resonators based on aluminum nitride thin films. Appl. Phys. Lett. 95, 103111 (2009).

[b15] Tortonese M., Barrett R. C. & Quate C. F. Atomic resolution with an atomic force microscope using piezoresistive detection. Appl. Phys. Lett. 62, 834–836 (1993).

[b16] Harley J. A. & Kenny T. W. High-sensitivity piezoresistive cantilevers under 1000 Å thick. Appl. Phys. Lett. 75, 289–291 (1999).

[b17] Ivaldi P. *et al.* 50 nm thick AlN film-based piezoelectric cantilevers for gravimetric detection. J. Micromech. Microeng. 21, 085023 (2011).

[b18] Schwalb C. H. *et al.* A tunable strain sensor using nanogranular metals. Sensors 10, 9847–9856 (2010). 22163443 10.3390/s101109847 PMC3231023

[b19] Utke I., Hoffmann P. & Melngailis J. Gas-assisted focused electron beam and ion beam processing and fabrication. J. Vac. Sci. Technol. B 26, 1197–1276 (2008).

[b20] Brenning H. T. a. *et al.* A single electron transistor on an atomic force microscope probe. Nano Lett. 6, 937–941 (2006). 16683829 10.1021/nl052526t

[b21] Koppinen P. J., Lievonen J. T., Ahlskog M. & Maasilta I. J. Strain sensing with submicron Al-AlO(x)-Al tunnel junctions. Rev. Sci. Instrum. 81, 023901 (2010). 20192503 10.1063/1.3298582

[b22] Huth M. Granular metals: from electronic correlations to strain-sensing applications. J. Appl. Phys. 107, 113709 (2010).

[b23] Zhang J. & Shklovskii B. Density of states and conductivity of a granular metal or an array of quantum dots. Phys. Rev. B 70, 115317 (2004).

[b24] Beloborodov I. S., Lopatin A. V., Vinokur V. M. & Efetov K. B. Granular electronic systems. Rev. Mod. Phys. 79, 469–518 (2007).

[b25] Sachser R., Porrati F., Schwalb C. H. & Huth M. Universal conductance correction in a tunable strongly coupled nanogranular metal. Phys. Rev. Lett. 107, 206803 (2011). 22181756 10.1103/PhysRevLett.107.206803

[b26] Sachser R., Reith H., Huzel D., Winhold M. & Huth M. Catalytic Purification of directly written nanostructured pt microelectrodes. ACS Appl. Mater. Interfaces 6, 15868–15874 (2014). 25111450 10.1021/am503407y

[b27] Fowlkes J. D. *et al.* Electron nanoprobe induced oxidation: a simulation of direct-write purification. Phys. Chem. Chem. Phys. 17, 18294–18304 (2015). 26058775 10.1039/c5cp01196e

[b28] Adams J. D. *et al.* Analysis of local deformation effects in resistive strain sensing of a submicron-thickness AFM cantilever. in *Proceedings of Conference on Smart Sensors, Actuators, and MEMS VI*, 876327 (Grenoble, France, 2013).

[b29] Zheng M. *et al.* Strain sensors based on chromium nanoparticle arrays. Nanoscale 6, 3930–3933 (2014). 24132035 10.1039/c3nr04135b

[b30] Yi L. *et al.* Ultrasensitive strain gauge with tunable temperature coefficient of resistivity. Nano Res. 9, 1346–1357 (2016).

[b31] Antognozzi M. *et al.* A new detection system for extremely small vertically mounted cantilevers. Nanotechnology 19, 384002 (2008). 21832562 10.1088/0957-4484/19/38/384002

[b32] Sanii B. & Ashby P. D. High Sensitivity deflection detection of nanowires. Phys. Rev. Lett. 104, 147203 (2010). 20481957 10.1103/PhysRevLett.104.147203

[b33] Kolb F. *et al.* Variable tunneling barriers in FEBID based PtC metal-matrix nanocomposites as a transducing element for humidity sensing. Nanotechnology 24, 305501 (2013). 23818049 10.1088/0957-4484/24/30/305501

[b34] Huth M., Kolb F. & Plank H. Dielectric sensing by charging energy modulation in a nano-granular metal. Appl. Phys. A 117, 1689–1696 (2014).

[b35] Anetsberger G. *et al.* Near-field cavity optomechanics with nanomechanical oscillators. Nat. Phys. 5, 909–914 (2009).

[b36] Lee S. *et al.* A strain-absorbing design for tissue–machine interfaces using a tunable adhesive gel. Nat. Commun. 5, 5898 (2014). 25523614 10.1038/ncomms6898

[b37] Cui Y., Wei Q., Park H. & Lieber C. M. Nanowire nanosensors for highly sensitive and selective detection of biological and chemical species. Science 293, 1289–1292 (2001). 11509722 10.1126/science.1062711

