# Supplementary material for: Direct-write nanoscale printing of nanogranular tunnelling strain sensors for sub-micrometre cantilevers
Source: Nat Commun. 2016 Sep 26;7:12487. doi: 10.1038/ncomms12487 (PMC5052671; doi:10.1038/ncomms12487)
Supplement: Supplementary Information — Supplementary Figures 1-9, Supplementary Tables 1-2, Supplementary Notes 1-8 and Supplementary References [file ncomms12487-s1.pdf]

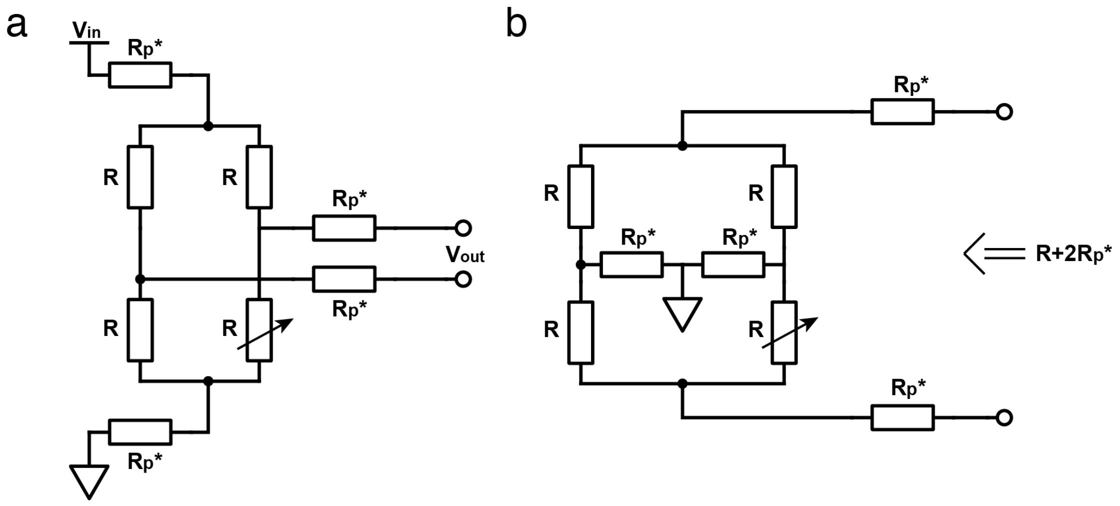

Supplementary Figure 1 | Influence of parasitic resistances on the signal-to-noise ratio. **a,** Schematic of the Wheatstone bridge readout **b,** Equivalent resistance circuit for Johnson noise estimation

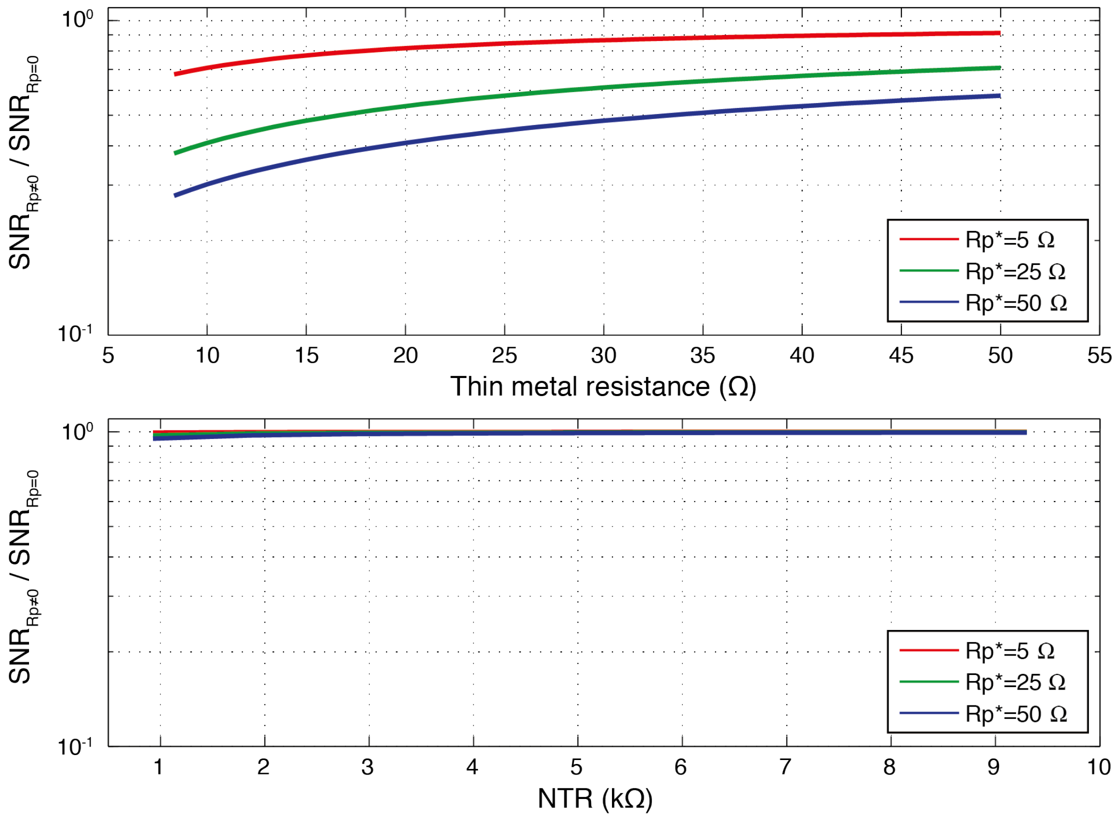

Supplementary Figure 2 | Signal-to-noise ratio loss in nanogranular tunneling resistor and thin gold film readout due to parasitic resistances: a, SNR(Rp≠0)/SNR(Rp=0) for a thin gold film resistor. **b,** SNR(Rp≠0)/SNR(Rp=0) for a nanogranular tunneling resistor (NTR), for several different values of connecting trace parasitic resistance.

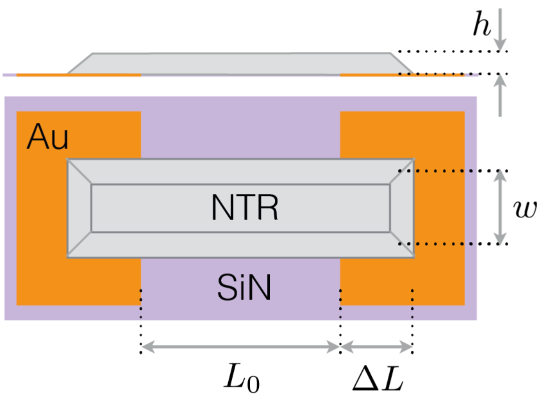

Supplementary Figure 3 | Geometry of Pt(C) deposits between Au electrodes. Gap lengths L_0 are 40 nm, 80 nm, 100 nm, 200 nm and 500 nm. Average width w of conically shaped deposits is 500 nm. Height is approximately 80 nm. The length of the deposits on the Au electrodes ΔL is 1.2 µm.

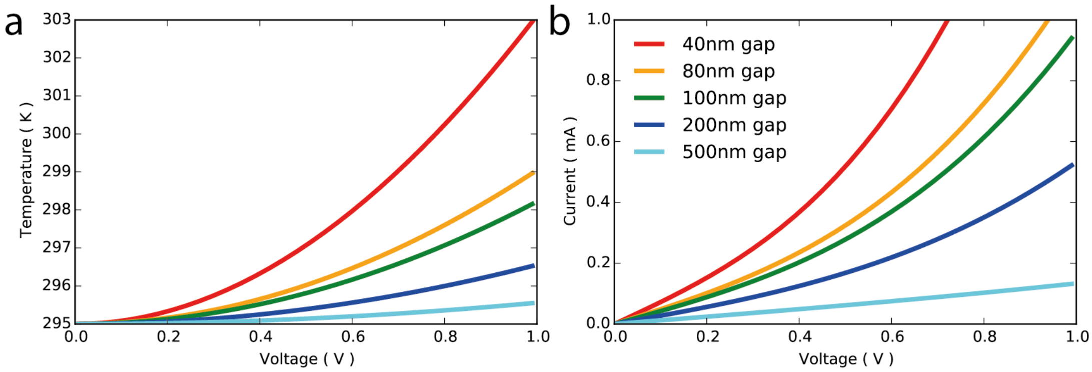

Supplementary Figure 4 | Heating influence - matching the theoretical calculations to the to the measured data: a, calculated temperature change of samples with various gap length and **b,** associated measured I(V)-characteristics.

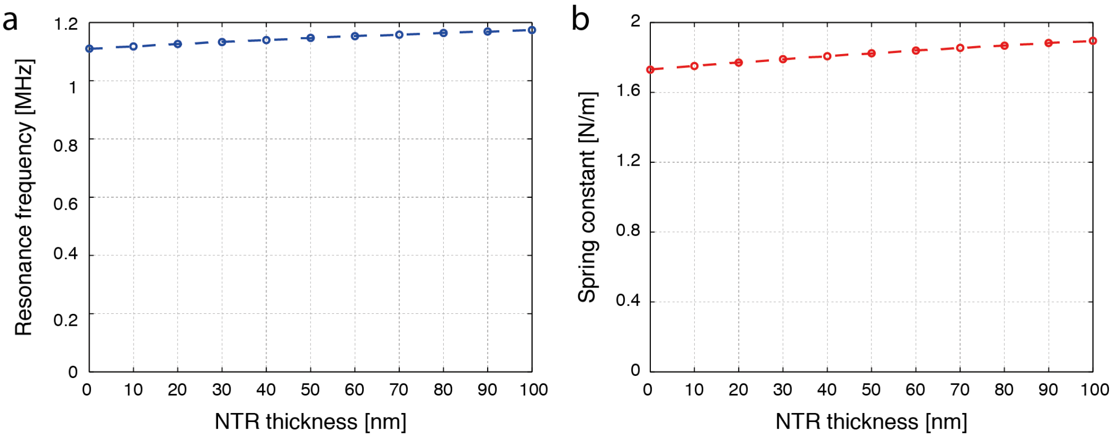

Supplementary Figure 5 | Results of finite element analysis simulation showing how increasing the nanogranular tunneling resistor thickness affects the cantilever: a, resonance frequency and **b,** spring constant. We performed finite element analysis (FEA) simulation to determine how deposition of full bridge of nanogranular tunneling resistors (NTRs) affects cantilever resonance frequency and spring constant. We simulated SiN cantilever having size 20×8×0.3 µm³ (similar as the one presented in Figure 4a in the paper) where we changed thickness of NTR sensors from 0 to 100 nm. The presented results show very small deviation in both resonance frequency and spring constant.

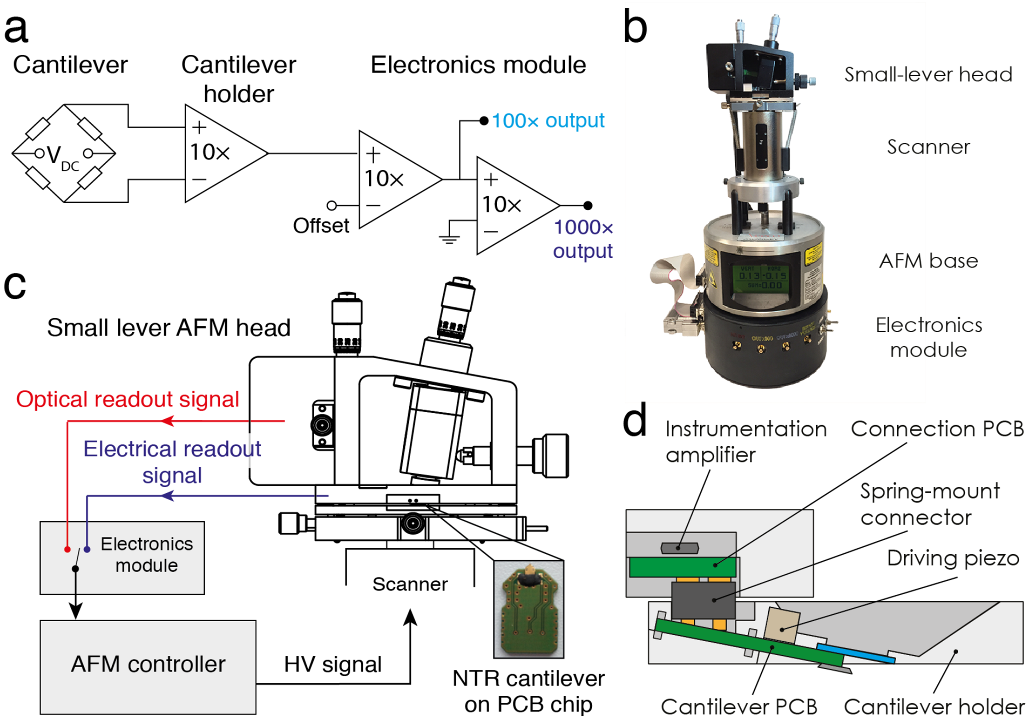

Supplementary Figure 6 | Atomic force microscopy measurement setup. a, Schematic of the electronic readout. **b,** atomic force microscopy (AFM) measurement setup, **c,** schematic of the AFM measurement setup for both optical and electrical readout **d,** schematic of the cantilever holder

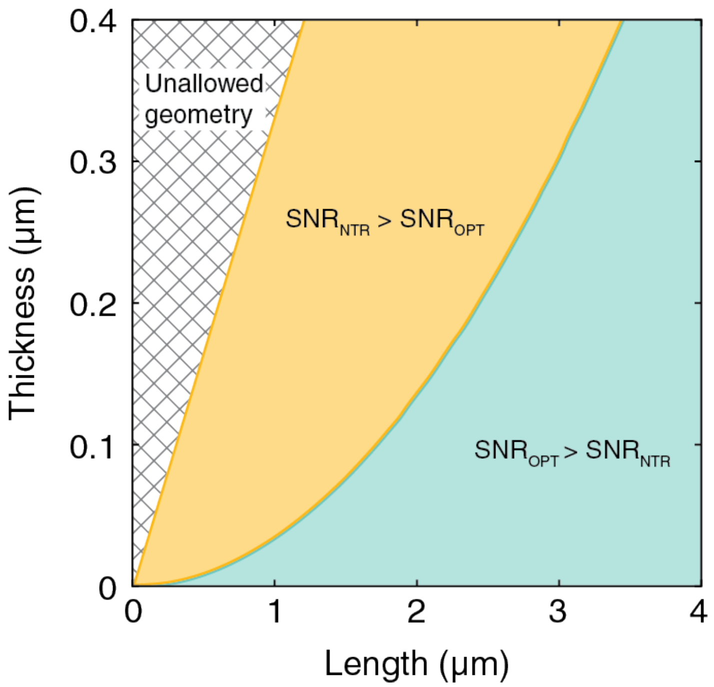

Supplementary Figure 7 | Parameter space of cantilever length and thickness comparing the signal-to-noise ratio of optical and nanogranular tunneling resistor deflection detection. The region marked in green indicates expected geometries where optical sensing outperforms nanogranular tunneling resistor (NTR) sensing, if optical detection were practical to implement for these cantilever geometries. The region marked in yellow indicates where NTR sensing is expected to outperform optical sensing. Unallowed geometries are such that the cantilever thickness would exceed the width.

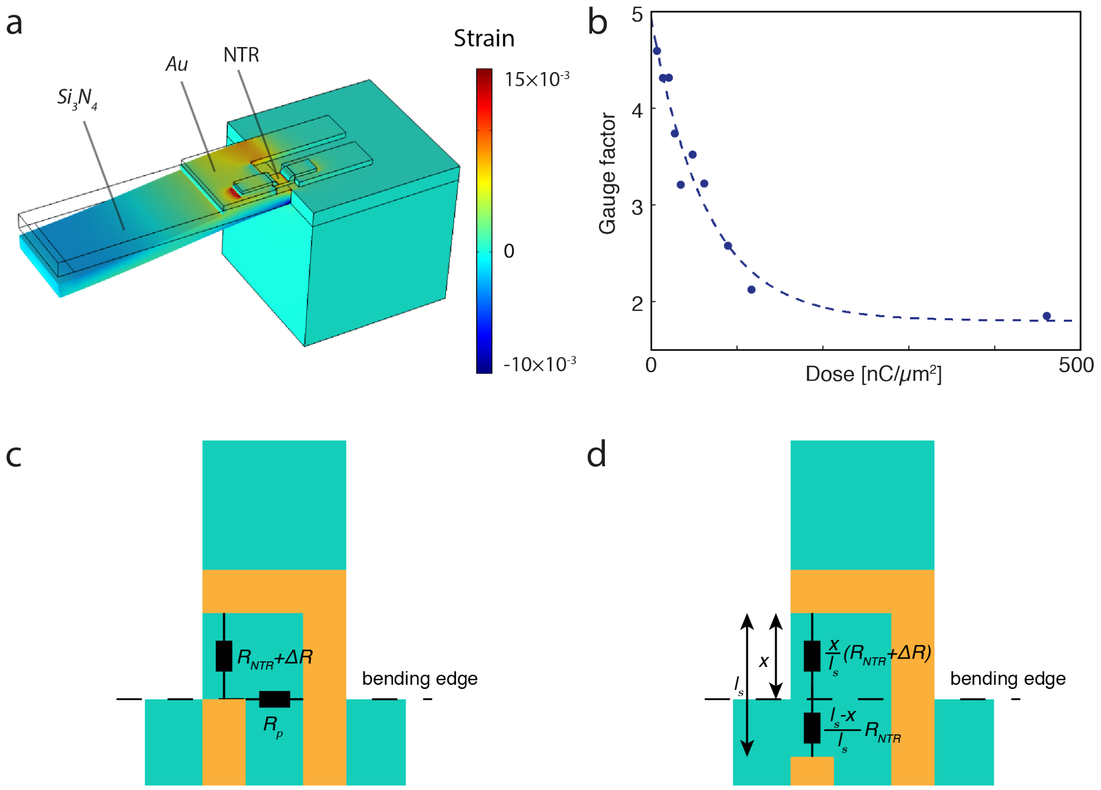

Supplementary Figure 8 | Estimation of gauge factor for small sized nanogranular tunneling resistor. a, Strain distribution in the structure simulated in COMSOL for 50 nm cantilever deflection. Cantilever size: 1.5×0.5×0.1 μm³, active nanogranular tunneling resistor (NTR) size: 150×100×20 nm. **b,** estimated gauge factors from simulated strain in the structure and measured change of the relative resistance. **c+d,** possible scenarios for the reduction of the apparent NTR gauge factor: current flow directly from one electrode to another through parasitic resistance $\boldsymbol{R_P}$ (c) and misalignment of the gold contact electrodes, for which only part of the NTR acting as strain sensor (d).

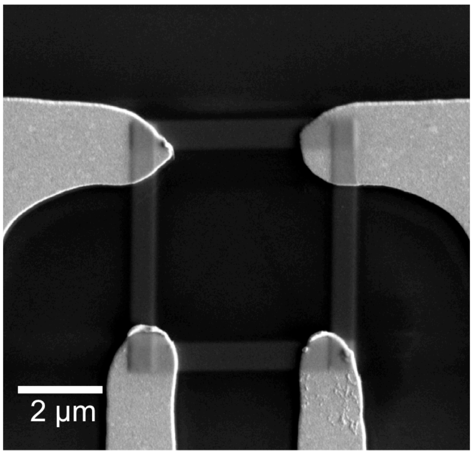

Supplementary Figure 9 | Scanning electron microscopy image of the nanogranular tunneling resistors deposited on a cantilever using the semi-automated deposition process.

Supplementary Table 1 | Parameters used for calculation of the expected SNR performance of thin gold film sensor versus NTR sensor

Parameter	Thin gold film	NTR
Resistor shape	U shaped	Cuboid
Length l_s [μm]	0.1 – 6<br>(arm length is $l_s/2$)	0.05 – 0.5
Width w_s [μm]	0.2	0.6
Thickness t_s [nm]	30	30
Gauge factor κ[1–3]	4.5	6
Resistivity ρ [Ω m]	5×10^{-8}	3.37×10^{-4}
Maximum current density J_{max} [A/m^2]	Limited by electromigration: 1×10^{11}	Limited by nonlinearity in conductivity:<br>E_{max}=1.5 MV/m, $J_{max}=E_{max}/\rho$

Supplementary Table 2 | Parameters used in calculation of the expected relative SNR performance of NTR sensors versus optical deflection detection.

Parameter	Value
E (silicon nitride)	250 GPa
r (silicon nitride)	3100 kg/m^3
h (air)	18.6×10^{-6} Pa·s
M (air)	0.02897 kg/mol
k	8
R_{NTR}	300 Ω
V_S	0.4 V
l_g	40 nm
a	2 mm
s	1 cm
l (for calculation of n_{OPT} only)	20 μm
S	0.45 A/W
P	0.5 mW

Supplementary Note 1: influence of parasitic resistances on the signal-to-noise ratio (SNR)

One suggested method for the deflection readout of small sized cantilevers is resistive strain sensing using thin gold films[4]. However, as the size of the cantilever is decreased, the resistance of the thin gold film resistor decreases as well. In the measurement setup there will always exist some parasitic resistances of the connection lines. When these resistances become on par with the resistance of the strain sensing resistor, the SNR of the readout will start to decrease significantly. One benefit of the NTRs is that they can be deposited with sub-micron dimensions while their resistance can be tuned by post irradiation, hence always keeping the resistance in an optimal range.

In order to compare the SNR loss due to parasitic resistances in thin gold films and NTR deflection readout, we performed a case study. We assume that the change of the resistance is measured using a Wheatstone bridge, with one active resistor positioned on the cantilever and three passive resistors. We will denote the total parasitic resistance of each trace connecting the bridge as R_P^* (see Supplementary Figure 1a). Parasitic resistances of the connection lines in the bridge itself are neglected as they can be designed to be fairly small.

For the ease of writing, we define $R_P = 2R_P^*$. We would like to maximize the input bridge voltage V_{in} in order to get a higher output signal. The maximum input voltage that we can apply is defined by the maximum allowed current through the resistor I_{max} as $V_{in,max} = 2(R + R_P)I_{max}$, where R is resistance of the bridge resistors. In this case, for a certain cantilever deflection δ, the maximum achievable output voltage of the circuit is

$$V_{out} = \kappa \cdot \frac{3\left(l - \frac{l_s}{2}\right)(t + t_s)}{2l^3} \cdot \delta \cdot \frac{I_{max}R}{2} \tag{1}$$

where l and t are the cantilever length and thickness; l_s and t_s represent the length and thickness of the sensing resistor and κ is sensor gauge factor.

In order to estimate the SNR of the readout, we are considering the intrinsic noises of the circuit resistors. For simplicity, we will not take into account the low frequency $1/f$ noise as we are only considering the case where resistors are used for strain sensing at higher frequencies, with band pass filtering around the working frequency. In this case the overall circuit noise comes from the circuit Johnson noise:

$$V_J = \sqrt{4k_BTB(R + R_P)} \tag{2}$$

where k_B, T and B represent Boltzmann constant, temperature in Kelvins and measurement bandwidth, respectively. This formula results from the fact that V_{in} is an AC ground, so the equivalent resistance seen from the output ports of the bridge is $R + 2R_P^* = R + R_P$ (see Supplementary Figure 1b).

The SNR of the readout for a given cantilever deflection δ can be calculated as

$$SNR = \frac{V_{out}}{V_J} = \kappa \cdot \frac{3\left(l - \frac{l_s}{2}\right)(t + t_s)}{4l^3} \cdot \frac{I_{max}}{\sqrt{4k_B TB}} \cdot \frac{R}{\sqrt{R + R_P}} \cdot \delta \qquad (3)$$

Case study

The cantilever used in our calculations is a silicon nitride cantilever ($E = 250\,GPa, \rho = 3100\,kg/m^3$), having dimensions of $3{\times}1{\times}0.1\,\mu m^3$. The measurement bandwidth is set to $500\,kHz$ and the point load induced deflection of the cantilever is set to $\delta = 1\,nm$. Calculations were made for three values of the connection lines parasitic resistance $R_P^* = 5\,\Omega, 25\,\Omega$ and $50\,\Omega$.

Two types of resistive strain sensors were compared: a U-shaped thin gold film resistor and a cuboid shaped NTR resistor. The assumed properties of each resistor are given in Supplementary Table 1. Properties for each resistor are chosen in a way to maximize its performance. The resistivity value chosen for the thin gold film corresponds to the resistivity value we measured for an evaporated 5/30 nm thick Cr/Au film. The irradiation dose used for NTR post deposition resistance tuning is $75\,nC/\mu m^2$.

Results obtained by the calculation are plotted in Supplementary Figure 2. SNR losses due to parasitic resistances are much larger for the case of the thin gold film resistor. This is because the maximum achievable resistance for the gold film resistor is around 50 Ω (when the gold trace goes along the whole cantilever length). By varying post deposition irradiation dose and resistor length, for chosen thickness and width of the sensor (Supplementary Table 1), the resistance of the NTR can be tuned from several 100s Ω to several 10s of kΩ.

Supplementary Note 2: explanation of the signal-to-noise ratio expression

In the paper equation (1), the signal-to-noise ratio is defined as

$$SNR = \frac{A_{max}}{n_J \cdot DS} \tag{4}$$

where A_{max} represents the peak of the cantilever thermomechanical noise spectral density at cantilever resonance frequency, n_J is the NTR noise spectral density at cantilever resonance frequency and DS is the deflection sensitivity of the measurement system.

A_{max} is given by

$$A_{max} = \sqrt{\frac{4}{3} \cdot \frac{4k_B T Q}{2\pi f_0 k}} \tag{5}$$

where k_B and T are Boltzmann constant and temperature; Q, k and f_0 are cantilever quality factor, spring constant and resonance frequency. The parameter 4/3 represents a correction factor due to a non-perfect harmonic oscillator behaviour of the cantilever and the fact that induced strain, rather than deflection is measured with strain-sensing element[5–7].

The NTR noise spectral density n_J[8] and the deflection sensitivity DS[9] are given by

$$n_J = \sqrt{4k_B T R} \tag{6}$$

$$DS = \frac{8}{3} \cdot \frac{1}{\kappa V_S} \cdot \frac{l^2}{t\left(1 - \frac{L}{2l}\right)} \tag{7}$$

where R is resistance of the NTR, V_S is supply voltage to the Wheatstone bridge, κ is gauge factor, L is NTR sensor length, and l and t are cantilever length and thickness. For simplicity, in the paper, the numerical constant 0.173 in the SNR expression is calculated from all other numerical constants.

Supplementary Note 3: I(V)-characteristics nonlinearity due to local heating effects

A local heating predominately results in changes of the conductivity of the NTR. We have derived the following analysis of the contribution of the heating and matched the theoretical explanation to the to the measured data.

We start with the geometry of the Pt(C) deposits with different gap sizes between Au electrodes, as shown below, in Supplementary Figure 3. We begin our analysis with the entropy continuity equation, as follows

$$\frac{dS}{dt} = \chi - I_S \tag{8}$$

where the entropy rate of change dS/dt in the deposit is given by the entropy production rate χ due to the current flow I and the entropy current I_S leaving the deposit towards the Au electrodes and substrate. We have furthermore

$$\chi = \frac{V^2}{R}\frac{1}{T} = V^2\sigma(T)\frac{wh}{L_0}\frac{1}{T} \tag{9}$$

where V, R, $\sigma(T)$ and T denote the applied voltage, the sample resistance, the sample conductivity and temperature, respectively. For the entropy current we note

$$I_S = \sigma_S\frac{A_{eff}}{L_{eff}}(T - T_0). \tag{10}$$

T_0 is the bath temperature referring to the Au electrodes and substrate. We set $T_0 = 295\ K$. σ_S is the entropy conductivity which we assume to be approximately temperature independent, for simplicity. For the effective area A_{eff} and length L_{eff} we set in reasonable approximation

$$A_{eff} = w(L_0 + 2\Delta L) \quad \text{and} \quad L_{eff} = \frac{h}{2}. \tag{11}$$

Under steady-state conditions $dS/dt = 0$ we obtain immediately from Eq. (8)

$$V^2\frac{\sigma(T)h}{L_0T} = \frac{2\sigma_s(L_0 + 2\Delta L)}{h}(T - T_0). \tag{12}$$

Solving for T we finally have

$$T_{1/2} = \frac{T_0}{2} \pm \sqrt{\frac{T_0^2}{4} + \frac{1}{2}\frac{\sigma(T)}{\sigma_S}\frac{h^2}{L_0(L_0 + 2\Delta L)}V^2}, \tag{13}$$

with T_1 being physically relevant for calculating the steady-state temperature of the deposit due to energy dissipation during current flow.

The temperature-dependent conductivity of the deposits we obtain from a wealth of independent measurements in the temperature range from 295 K down to about 2 K. In the present case, the deposits were irradiated with $74\ nC/\mu m^2$ which we take into account. Also, from our data, presented in paper Figure 3e, we can directly obtain the conductance at T_0 by linear extrapolation of the I(V)-characteristics.

The entropy conductivity σ_S or, alternatively, heat conductivity $\lambda = \sigma_S T$ is not readily available. From recent theoretical work on nano-granular metals in the strong-coupling limit[10] we estimate

$$\sigma_S \approx 2\times10^{-2}\ W/m\ K^2 \quad (14)$$

For the sample with $L_0 = 500\ nm$ gap size and adapt it accordingly for the other samples depending on their respective electrical conductivity, as $\sigma_S \propto \sigma$ for granular metals in the strong-coupling regime[10]. The results of our calculation providing an estimate of the samples' temperature for different gap lengths, as well as for the I(V)-characteristics are shown in Supplementary Figure 4. Considering the rather crude model used for the calculation, we can state a rather good correspondence between the simulation and the measured data. We conclude that the dominating factor for the non-linear behavior of the I(V)-characteristics at small gap sizes is a heating effect.

Supplementary Note 4: Influence of the FEBID sensor deposition on the cantilever dynamics

We investigated the effect of the sensor mass and stiffening on the cantilever resonance frequency and spring constants using finite element analysis (FEA) The obtained results are shown in Supplementary Figure 5. Even when going to extreme sensor thicknesses of 30% of the cantilever thickness, the effect is only a ~10% shift, in both the resonance frequency and spring constant. We chose finite element analysis rather than actual experiments since the spread in mechanical properties of the base cantilevers due to the typical fabrication tolerances is much larger than the effect of the added mass.

Supplementary Note 5: AFM measurement setup and procedure

AFM imaging with NTR cantilevers was performed using a custom-made electronics setup and custom-made AFM head (see Supplementary Figure 6a-c). Details regarding the design of the AFM head are previously published in reference[11]. The custom-made AFM head allowed us to compare the self-sensing performance of the small 20×8 μm^2 sized NTR cantilevers to optical beam deflection (OBD) readout. A custom-made fluid-sealed cantilever holder allowed for imaging in both air and liquid environments (see Supplementary Figure 6d). All connection lines on the cantilever and the PCB chip were fluid isolated, except for the NTRs themselves, which were in direct contact with fluid.

The electrical readout of the NTR cantilevers consisted of a full Wheatstone bridge of NTR resistors on the cantilever and subsequent amplification stages (Supplementary Figure 6a). The first amplification stage, a low noise instrumentation amplifier (AD8250), was positioned in the AFM head close to the cantilever sensor to reduce noise and stray capacitances from the Wheatstone bridge electrical connections. A high precision voltage reference (ADR130) biased the bridge. The subsequent amplification stages were located in an electronics module below the standard Bruker MultiMode AFM base and designed to intersect between the Bruker Nanoscope controller and the Bruker MultiMode AFM base (see Supplementary Figure 6b-c).

For AFM measurements the DC offset of the Wheatstone bridge was first zeroed. The electrical and optical deflection signals were verified by a frequency sweep of the tapping excitation piezo; the appropriate working frequency was further confirmed via a thermal tune using optical deflection detection. Finally, appropriate cantilever free and set point oscillation amplitudes, gains and other imaging parameters were chosen.

Supplementary Note 6: Estimate of cantilever length scale where NTRs outperform optical detection

Here we consider as the signal to noise ratio (SNR) the total thermomechanical peak height p_{tm} versus the baseline noise floor. Both NTR Johnson noise and optical beam deflection photodetector shot noise may be considered as white noise sources with noise floor n and ripple δ. The total peak height of the thermomechanical peak is the square root of the sum of the squared thermomechanical noise t_{tm} and the white noise

$$p_{tm} = \sqrt{t_{tm}^2 + n^2}. \tag{15}$$

The SNR is then the height of the peak above the baseline, divided by the ripple in the noise floor

$$SNR = \frac{p_{tm} - n}{\delta} = \frac{\sqrt{\left(\frac{t_{tm}}{n}\right)^2 + 1} - 1}{\frac{\delta}{n}}. \tag{16}$$

Assuming both optical and NTR deflection detection are measured with similar conditions (measurement bandwidth, averaging time) the ratio δ/n can be considered equal between optical and NTR detection; therefore the ratio of SNRs can be expressed as

$$\frac{SNR_{NTR}}{SNR_{OPT}} = \frac{\sqrt{\left(\frac{t_{tm}^{NTR}}{n_{NTR}}\right)^2 + 1} - 1}{\sqrt{\left(\frac{t_{tm}^{OPT}}{n_{OPT}}\right)^2 + 1} - 1}. \tag{17}$$

In length units the Johnson noise density of the NTR sensor may be estimated from the Johnson noise density of the resistor scaled by the deflection sensitivity of the NTR Wheatstone bridge, which for small deflections may be expressed as[8]

$$\frac{dV_G}{d\Delta z} = \frac{3\kappa V_s \left(1 - \frac{l_g}{2l}\right) t}{8l^2} \tag{18}$$

where V_G is the Wheatstone bridge voltage, z is the cantilever end deflection, κ the NTR gauge factor, V_s the Wheatstone bridge supply voltage, l_g the length between contacts of the NTR sensor, l the cantilever length, and t the cantilever thickness. The Johnson noise of the NTR element is then

$$n_{NTR} = \frac{8l^2\sqrt{4k_B T R_{NTR}}}{3\kappa V_s \left(1 - \frac{l_g}{2l}\right) t} \tag{19}$$

where k_B is the Boltzmann constant, T the temperature of the sensor (assumed 298 K), and R_{NTR} the resistance of the NTR element. The noise floor of the optical beam deflection system is given by[12]

$$n_{OPT} = \frac{la}{3s}\sqrt{\frac{2e}{SP}} \quad (20)$$

where a is the size of the laser spot on the photodetector, s the distance from the cantilever to the photodetector, e the electron charge, S the photosensitivity of the photodetector, and P the laser power at the photodetector. For practical geometrical parameters (see Supplementary Table 2), this noise floor is about 50 fm/√Hz at 0.5 mW of laser power at the photodetector. The thermal peak for the optical deflection measurement is given by

$$t_{tm}^{OPT} = \sqrt{\frac{1.634 k_B T Q}{\pi k f_0}} \quad (21)$$

and for the NTR deflection by

$$t_{tm}^{NTR} = \sqrt{\frac{2 k_B T Q}{\pi k f_0}}. \quad (22)$$

The difference is due to the optical system measuring an angle change as opposed to a displacement[5]. The cantilever resonance frequency f_0, spring constant k and quality factor Q are calculated from

$$f_0 = 0.1615\frac{t}{l^2}\sqrt{\frac{E}{\rho}}, \qquad k = \frac{Ewt^3}{4l^3}, \qquad Q = \frac{4\rho t w f_0}{6\eta + 3w\sqrt{\eta \frac{M}{RT}\pi f_0 p}} \quad (23)$$

where E is the cantilever Young's modulus, ρ the cantilever density, w the cantilever width, η the viscosity of the surrounding medium (air), M the molecular mass of air, R the universal gas constant and p the pressure of the surrounding medium (assumed 1 atm).

In order to simplify the parameter space, we assume a cantilever planar shape such that $l = 3w$. Supplementary Table 2 lists values of the relevant parameters. Supplementary Figure 7 illustrates the regions where $\mathrm{SNR_{OPT}} > \mathrm{SNR_{NTR}}$ and where $\mathrm{SNR_{NTR}} > \mathrm{SNR_{OPT}}$. Starting below 3 μm length, there exists a broad range of cantilever geometries where the expected NTR signal to noise ratio is larger than the optical signal to noise ratio. This NTR performance advantage becomes especially prominent at submicron cantilever lengths, in addition to the extreme practical limitations of optically detecting these cantilevers.

Supplementary Note 7: Apparent gauge factor of the smallest NTR sensors

In order to estimate gauge factors of the NTR structure deposited on the 1.5×0.5×0.1 μm silicon nitride cantilever, a finite element analysis simulation was performed in COMSOL Multiphysics (see Supplementary Figure 8a). To calculate the strains in the structure, we estimated that the elastic modulus of the NTR is close to the Young's modulus of a diamond-like structure of amorphous carbon[13,14]. Therefore, in simulations we assumed the Young's modulus for NTR of 760 GPa.

For irradiation doses in the range from 0 – 500 nC/μm^2, estimated gauge factor values were in the range of 2 – 5 (see Supplementary Figure 8b). This range of values is lower than gauge factors measured for larger cantilevers and NTR structures. We suspect that this is due to several reasons:

1. Assumed values for the NTR Young's modulus might be off and we might have stronger stiffening effects of the cantilever.
2. If the NTR is too broad, a fraction of the current can directly flow from the left to the right electrode and not over the bending edge (see Supplementary Figure 8c). For this part the resistance does not change during bending of the cantilever.
3. If the gold contacts are not located at the bending edge but are slightly offset back on the chip body, the effective change of resistance ΔR is reduced to $x/l_s \cdot \Delta R$ (see Supplementary Figure 8d). This effect was incorporated in the COMSOL simulation. However it was difficult to estimate the exact position of the cantilever bending edge from the SEM cantilever image.

Supplementary Note 8: Semi-automated deposition system

The semi-automated deposition of the NTR sensor elements was realized in a dual-beam FEI Nova Nanolab 600 SEM containing the commercial software *Runscript™* that was used for implementation of the semi-automated process. The process consists of four main steps:

1. *Correction of z-height for the full wafer:* The user determines the coordinates (x, y, z) of three non-co-linear points on the wafer structure that are used for calculating the plane of the wafer. This step is done before the automation procedure is started. Afterwards the z-height for each NTR deposition can be corrected to ensure optimal working distance.
2. *Auto coarse approach of individual cantilever structure:* Using the wafer layout a coordinate map for each individual cantilever structure is created. Moving the SEM stage to the stored coordinates approaches each cantilever. Fine approach is performed by step (3).
3. *Image recognition of electrode structure and auto-alignment for each cantilever:* A reference image of the electrode structure is used for image recognition and auto-alignment of x- and y-position for each cantilever structure. In addition, an auto-focus procedure provided by Runscript™ is used prior to the NTR deposition.
4. *NTR sensor element deposition:* The pattern files that contain all parameters for NTR deposition are created. The z-height is corrected (1) and the auto-alignment procedure (2) is performed. The gas injection needle introducing the precursor gas is inserted and the deposition process is started. All four NTRs on each cantilever are deposited in parallel mode to minimize drifting of the electron beam.

Supplementary Figure 9 shows an SEM image of a cantilever equipped with four NTRs which were deposited using the semi-automated process. A movie of the semi-automated process is shown in Supplementary Movie 2.

Supplementary References

1. Ibbotson, R. H., Dunn, R. J., Djakov, V., Ferrigno, P. K. & Huq, S. E. Polyimide microcantilever surface stress sensor using low-cost, rapidly-interchangeable, spring-loaded microprobe connections. *Microelectron. Eng.* **85,** 1314–1317 (2008).
2. Thaysen, J., Yal inkaya, A. D., Vettiger, P. & Menon, A. Polymer-based stress sensor with integrated readout. *Journal of Physics D: Applied Physics* **35,** 2698–2703 (2002).
3. Jen, S. U., Yu, C. C., Liu, C. H. & Lee, G. Y. Piezoresistance and electrical resistivity of Pd, Au, and Cu films. *Thin Solid Films* **434,** 316–322 (2003).
4. Li, M., Tang, H. X. & Roukes, M. L. Ultra-sensitive NEMS-based cantilevers for sensing, scanned probe and very high-frequency applications. *Nat. Nanotechnol.* **2,** 114–120 (2007).
5. Butt, H.-J. & Jaschke, M. Calculation of thermal noise in atomic force microscopy. *Nanotechnology* **6,** 1–7 (1995).
6. Ohler, B. Cantilever spring constant calibration using laser Doppler vibrometry. *Rev. Sci. Instrum.* **78,** 63701 (2007).
7. Hansen, O. & Boisen, A. Noise in piezoresistive atomic force microscopy. *Nanotechnology* **10,** 51–60 (1999).
8. Schwalb, C. H. *et al.* A tunable strain sensor using nanogranular metals. *Sensors* **10,** 9847–9856 (2010).
9. Doll, J. C. & Pruitt, B. L. Design of piezoresistive versus piezoelectric contact mode scanning probes. *J. Micromechanics Microengineering* **20,** 95023 (2010).
10. Beloborodov, I. S., Lopatin, A. V, Hekking, F. W. J., Fazio, R. & Vinokur, V. M. Thermal transport in granular metals. *Europhys. Lett.* **69,** 435–441 (2005).
11. Adams, J. D. *et al.* High-speed imaging upgrade for a standard sample scanning atomic force microscope using small cantilevers. *Rev. Sci. Instrum.* **85,** 93702 (2014).
12. Fukuma, T. & Jarvis, S. P. Development of liquid-environment frequency modulation atomic force microscope with low noise deflection sensor for cantilevers of various dimensions. *Rev. Sci. Instrum.* **77,** 43701 (2006).
13. Schultrich, B., Scheibe, H.-J., Grandremy, G. & Schneider, D. Elastic Modulus of Amorphous Carbon Films. *Phys. Status Solidi* **145,** 385–392 (1994).
14. Cho, S., Chasiotis, I., Friedmann, T. a & Sullivan, J. P. Young's modulus, Poisson's ratio and failure properties of tetrahedral amorphous diamond-like carbon for MEMS devices. *J. Micromechanics Microengineering* **15,** 728–735 (2005).
